# Designing libraries for pooled CRISPR functional screens of long noncoding RNAs

**DOI:** 10.1007/s00335-021-09918-9

**Published:** 2021-09-17

**Authors:** Carlos Pulido-Quetglas, Rory Johnson

**Affiliations:** 1 Department of Medical Oncology, Inselspital, Bern University Hospital, University of Bern, 3010 Bern, Switzerland; 2 Department for BioMedical Research, University of Bern, 3008 Bern, Switzerland; 3 Graduate School of Cellular and Biomedical Sciences, University of Bern, 3012 Bern, Switzerland; 4 School of Biology and Environmental Science, University College Dublin, Dublin, D04 V1W8 Ireland; 5 Conway Institute for Biomolecular and Biomedical Research, University College Dublin, Dublin, D04 V1W8 Ireland

## Abstract

Human and other genomes encode tens of thousands of long noncoding RNAs (lncRNAs), the vast majority of which remain uncharacterised. High-throughput functional screening methods, notably those based on pooled CRISPR-Cas perturbations, promise to unlock the biological significance and biomedical potential of lncRNAs. Such screens are based on libraries of single guide RNAs (sgRNAs) whose design is critical for success. Few off-the-shelf libraries are presently available, and lncRNAs tend to have cell-type-specific expression profiles, meaning that library design remains in the hands of researchers. Here we introduce the topic of pooled CRISPR screens for lncRNAs and guide readers through the three key steps of library design: accurate annotation of transcript structures, curation of optimal candidate sets, and design of sgRNAs. This review is a starting point and reference for researchers seeking to design custom CRISPR screening libraries for lncRNAs.

## Introduction

The number of annotated long noncoding RNA (lncRNA) genes has grown dramatically in the past decade thanks to next-generation sequencing (NGS). However, our ability to functionally characterise these genes has failed to keep pace, meaning that the vast majority of lncRNAs are of unknown biological or disease relevance (Ma et al. 2019). Into this gap has stepped CRISPR-Cas genome editing, and to a lesser extent other forms of pooled and arrayed screening, which together promise to mine this large unexplored genetic space and reveal new biological players and disease targets. The design of screening libraries is a foundation for such studies and is the focus of this Review.

Although the majority of lncRNAs remain uncharacterised, several hundred have already been linked to diseases or cell functions (Kung et al. 2013; Lekka and Hall 2018). Examples are MALAT1 and SAMMSON, which promote tumorigenesis in vitro and in vivo through relatively well-defined molecular mechanisms (Gutschner et al. 2013; Leucci et al. 2016). Somatic mutations and expression dysregulation of genes encoding these lncRNAs are observed in tumours, in addition to other clinical evidence such as expression correlation with patient survival (Vendramin et al. 2018; Chen et al. 2018; Vancura et al. 2021). This link to disease has raised considerable interest in lncRNAs as targets for precision RNA therapeutics (Arun et al. 2018; Esposito et al. 2019; Fathi 2020; Xiong et al. 2021).

Their exceedingly large numbers make it essential to screen for functional lncRNAs using high-throughput methods. Unfortunately, technologies developed for protein-coding genes (PCGs) face a number of barriers when applied to lncRNAs. First among these is that RNA interference (RNAi) is often ineffective for lncRNAs, possibly due to the latter’s relative enrichment in the nucleus (Maamar et al. 2013; Stojic et al. 2016). RNAi also generates large numbers of off-target hits (Smith et al. 2017) and generating new RNAi arrayed libraries is expensive and involves complex robotics equipment. Another hindrance arises from the relatively poor state of lncRNA gene annotation (Uszczynska-Ratajczak et al. 2018), which has hindered the development of off-the-shelf arrayed or pooled screening libraries.

These challenges have recently been overcome by rapid developments in gene perturbation technologies. Two effective perturbation methods are now available, which together map a path from initial screening to therapeutic use in patients. Clustered regularly interspaced short palindromic repeats (CRISPR) afford versatile and highly scalable perturbations in the laboratory via direct targeting of the lncRNA gene itself (Shalem et al. 2015). Antisense oligonucleotides (ASOs) achieve co-transcriptional degradation, representing both a powerful experimental tool and effective therapeutic, but at relatively low throughputs (Gutschner et al. 2013; Meng et al. 2015). Both CRISPR and ASOs are relatively low cost, practical, and have low off-target rates (Smith et al. 2017; Yoshida et al. 2019). Nonetheless, each method has drawbacks that must be mitigated. For example, CRISPR can be economically scaled to high throughputs, but wild-type (WT) CRISPR-Cas9 causes double strand breaks (DSBs) in DNA, whose toxicity can lead to unintended consequences (Chapman et al. 2012). This and other CRISPR approaches are highly sensitive to gene annotation quality. On the other hand, ASOs are relatively more costly to synthesise and are incompatible with pooled screening, which together have largely prevented their use at high throughputs. Nevertheless, these technologies, particularly CRISPR, open the door to economic high-throughput functional screening of lncRNAs.

All screening projects, including CRISPR, require the careful design of libraries of perturbation constructs. A critical input for such designs is accurate gene maps or annotations (Uszczynska-Ratajczak et al. 2018). The effectiveness of CRISPR perturbations is highly sensitive to correct targeting to gene’s TSS (Sanson et al. 2018). Unfortunately, annotations for lncRNAs tend to suffer from several issues, making them a constraint in CRISPR screens. We will discuss these issues in more detail and outline solutions to maximise annotation quality.

When performing high-throughput experiments for lncRNAs, a critical question to address is the following: “Which genes will we target?”. Only a minority of genes are likely to be candidates in a given biological system, not least because the cell model will only express a small fraction of the total “lncRNA-ome” (Jiang et al. 2016; Seifuddin et al. 2020). For example, Cabili et al. demonstrated that 78% of lncRNAs are expressed in a tissue-specific manner (Cabili et al. 2011). Depending on the type and aim of the screen, the pool of gene candidates can vary considerably. Also, the cost of the screen increases with the number of targets analysed. Therefore, the selection of the smallest optimal set of candidates is important for the economic and scientific success of a project.

Finally, the user must design perturbation constructs with optimised on-target efficacy and minimal off-target effects. In the case of CRISPR, this corresponds to single guide RNAs (sgRNAs). Our understanding of the sequence and genomic features determining these properties continues to evolve.

This Review aims to highlight the main aspects of an optimal high-throughput lncRNA screen and will cover these principle topics: evolution of lncRNA screen technologies, and the three steps of screening library design: gene annotation, candidate selection, and sgRNA design.

## Functional screens for lncRNAs

### Long noncoding RNAs at the frontier of biology and medicine

LncRNAs are defined as RNA transcripts longer than 200 nt that are not translated into proteins (Derrien et al. 2012). In comparison with the total number of PCGs, relatively stable at ~ 19,000 annotated genes (Frankish et al. 2019), the total number of lncRNA gene loci in humans is still under discussion with estimations ranging from 16,000 up to 140,000 (Ma et al. 2019; Frankish et al. 2019). Among these, just ~ 2000 have been functionally characterised in any detail (Ma et al. 2019).

Although an unknown number of lncRNAs may represent non-functional transcriptional noise (Palazzo and Lee 2015; Doolittle 2018) or be misannotated since they encode a small peptide (Ingolia et al. 2011), numerous studies have ascribed convincing roles and detailed molecular mechanisms to a core set of widely studied genes. For example, studies have demonstrated important roles for lncRNAs in regulation of embryonic development (Kung et al. 2013), DNA damage repair (Thapar 2018), chromatin remodelling and modifications (Marchese et al. 2017) among others. Similarly, lncRNAs play clear roles in human diseases, such as neuronal disorders (Sparber et al. 2019), cardiac diseases (Turton et al. 2019) and most notably cancer, where hundreds of lncRNAs have been functionally linked to tumorigenesis and cancer hallmarks (Schmitz et al. 2016; Schmitt and Chang 2017). In the above cases, lncRNAs have met the levels of evidence required for identifying PCG function, including in some cases, phenotypes in knockout animals (Adriaens et al. 2016; Wen et al. 2016; Akay et al. 2019; Gao et al. 2020). As a result, growing attention has gathered on the possibility of using lncRNAs as therapeutic targets to treat human diseases (Schmitt and Chang 2017; Chen et al. 2021).

LncRNAs present unique challenges to researchers. Their lack of encoded peptides means that the longstanding and effective functional prediction tools for proteins are ineffective for lncRNAs (Johnsson et al. 2014). Numerous attempts have been made to bioinformatically predict lncRNA functions; however, these usually rely on indirect evidence (for example, expression correlation) (Guo et al. 2013; Jiang et al. 2015; Pyfrom et al. 2019) whose predictive power is uncertain (Perron et al. 2017). Another widely employed source of evidence for functionality is evolutionary conservation (Chodroff et al. 2010; Carlevaro-Fita et al. 2019; Ruiz-Orera and Albà 2019), but here too lncRNAs are challenging: they tend to display low levels of evolutionary conservation at the sequence level, even for confidently functional cases like Cyrano (Ulitsky et al. 2011), while many others have no identifiable orthologues at all (Vendramin et al. 2018; Washietl et al. 2014; Necsulea et al. 2014; Hezroni et al. 2015). These considerations drive the search for innovative approaches to prioritise lncRNAs for functional screens.

Amongst the tens of thousands of remaining lncRNAs, there is a lively debate as to what proportion represent functional genes vs transcriptional noise (Palazzo and Lee 2015; Doolittle 2018). Regardless of the outcome, it is likely that thousands of novel genes with important biological and disease roles remain to be discovered. The enormous number of lncRNAs, coupled to our present lack of means of predicting their function a priori, makes high-throughput functional screens the only viable route to identifying the subset of functional genes.

### Evolving tools for functional screening of lncRNAs

**Fig. 1:**
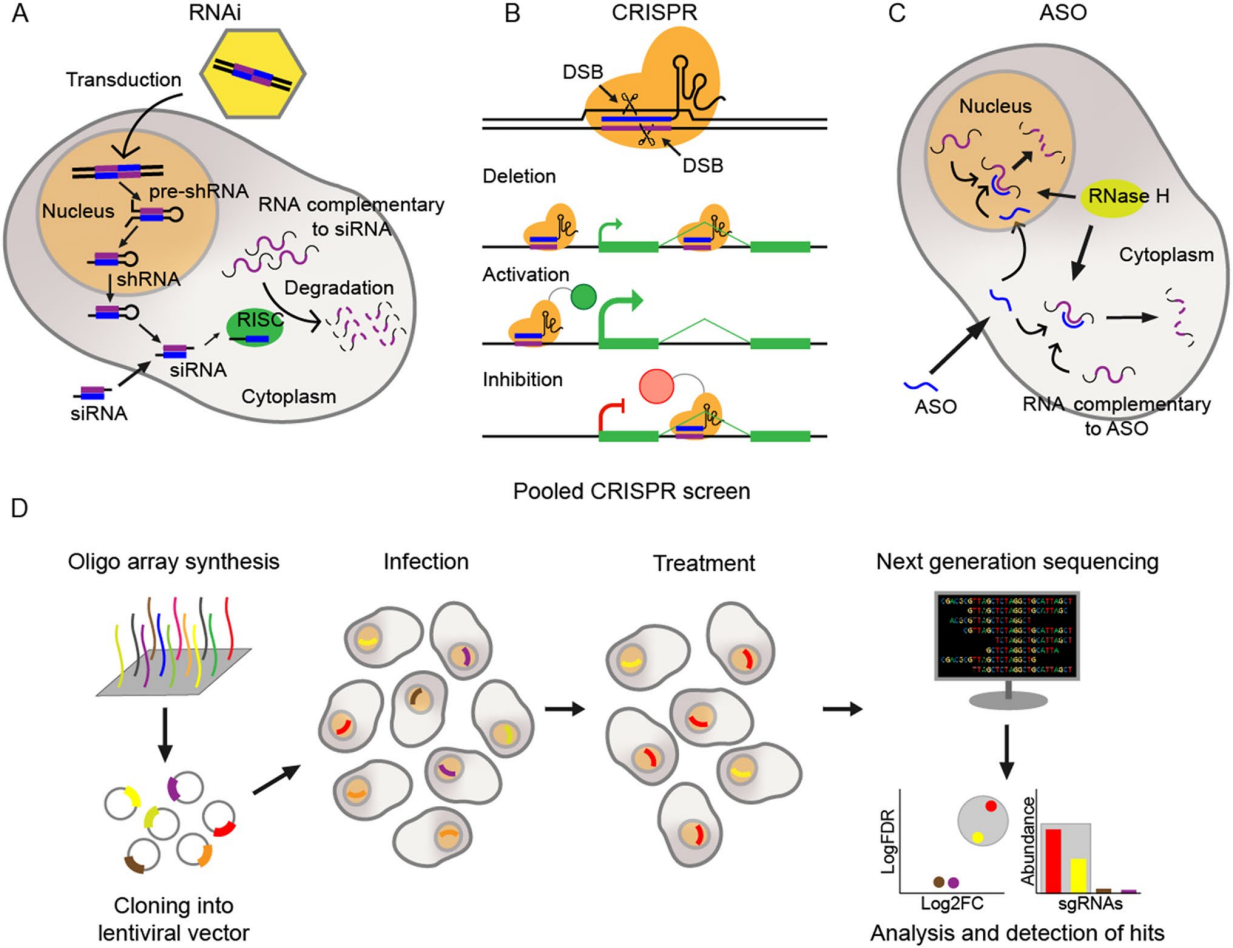
Perturbation methods and mechanisms. Molecular mechanism of **a** RNA interference, **b** various CRISPR perturbations (CRISPR/Cas9 activity occurs in the nucleus, while CRISPR/Cas13 activity can occur in either the nucleus or the cytoplasm), and **c** antisense oligonucleotides (ASOs). **d** The main steps of a pooled CRISPR screen

The large number of lncRNAs, coupled to our inability to predict their function, introduces the need for pooled functional screening approaches. Functional screening depends on two key factors: effective methods to perturb gene activity, and the degree to which such methods can be practically and economically scaled to high throughputs. The availability, or lack, of such techniques has dictated progress in lncRNA screening. Available perturbations fall into three principal types: RNA interference (RNAi) (effected by either small interfering RNAs or short hairpin RNAs); CRISPR-based perturbations; and ASOs (Fig. 1a–c). Here we introduce the principle perturbation methods for lncRNAs, then how they may be scaled to high throughputs by pooling.

#### Perturbation approaches: RNA interference

Early approaches to screen lncRNAs came from RNAi, which had a long history in PCG screening (Berns et al. 2004; Lord et al. 2008). RNAi depends on small (~ 22 bp) double-stranded RNAs that trigger degradation of complementary RNAs by the Argonaute family of proteins (Napoli et al. 1990; Fire et al. 1998; Cullen 2005). RNAi can be achieved by two distinct means: small interfering RNA (siRNA) and short hairpin RNA (shRNA). The two approaches differ in their delivery method (Fig. 1a), with important implications for screening. siRNA are chemically synthesised double-stranded oligonucleotides that must be delivered individually in an arrayed format, introducing the need for robotics and the generation of relatively expensive libraries (Rao et al. 2009). shRNAs are microRNA-like transcripts that are expressed as a single-stranded precursor, which folds into a hairpin structure and is recognised and processed into a double-stranded small RNA, similar to an siRNA (Elbashir et al. 2001; Caplen et al. 2001). shRNA genes may be delivered with a lentiviral plasmid, making them compatible with pooled screening (Sims et al. 2011). Given the topic of this Review, we here devote more space to shRNA; however, several important arrayed siRNA screens have been published (Whitehurst et al. 2007; Tiessen et al. 2019; Stojic et al. 2020).

shRNA has been used widely and successfully to screen PCGs, for example in Project Achilles (Tsherniak et al. 2017), although it is being rapidly supplanted by CRISPR (Bassik et al. 2009). The first pooled shRNA library for lncRNAs was designed to target 1280 intergenic mouse lncRNAs annotated in the ENSEMBL database (Lin et al. 2014). In a screen to identify lncRNAs involved in maintenance of pluripotency, the authors identified 20 hits, including TUNA. The size and focus of shRNA libraries can be adapted. For example, a larger library was designed for 3842 lncRNAs to identify those promoting proliferation of NIH3T3 mouse fibroblasts (Beermann et al. 2018). RNAi can also be adapted for in vivo experiments to study diseases. 120 lncRNAs were screened with a pooled shRNA library in a mouse model of acute myeloid leukemia, identifying 20 hits necessary for disease maintenance (Joaquina Delás et al. 2017).

Despite these successes, RNAi suffers from some notable drawbacks. First, RNAi perturbations often result in widespread unintended “off-target” repression of non-targeted genes (Smith et al. 2008). This is thought to occur as a result of the relatively short “seed” region through which RNAi target recognition takes place, resulting in large numbers of fortuitous matches in non-target genes (Birmingham et al. 2006; Sudbery et al. 2010). The outcome of this is observed phenotypic effects that arise independent of the intended target gene, i.e. false positives (Sudbery et al. 2010). The second principal drawback of RNAi is that it yields a variable and often low knockdown for lncRNAs (Lennox and Behlke 2016). The precise reasons for this remain unclear, and certainly many exceptions exist (Mondal et al. 2015; Gore-Panter et al. 2016), but it may be due to the preferential nuclear enrichment of lncRNAs (Carlevaro-Fita et al. 2019), whereas siRNA is more effective in the cytoplasm (Lennox and Behlke 2016; Zeng and Cullen 2002). A follow-on effect of this, is that it is suspected that even when successful lncRNA knockdown is observed, it may be the cytoplasmic RNA population that is preferentially affected, leaving nuclear activity intact (Maamar et al. 2013; Stojic et al. 2016). A more recent explanation came with the finding that siRNA requires translation to be effective, and hence only lncRNAs that are engaged by ribosomes will be impacted (Carlevaro-Fita et al. 2016; Biasini et al. 2021).

Thus, while a number of fruitful screens have been carried out for lncRNAs, both in pooled (Lin et al. 2014; Beermann et al. 2018; Joaquina Delás et al. 2017) and arrayed (Whitehurst et al. 2007; Tiessen et al. 2019; Stojic et al. 2020) formats, RNAi has not impacted the lncRNA field to the same extent as for PCGs, and researchers had to content themselves for many years with more conventional and low-throughput differential gene expression evidence as the starting point for identifying functional lncRNAs (Whitehurst et al. 2007; Lin et al. 2014).

#### Perturbation approaches: antisense oligonucleotides

A second perturbation approach worth mentioning is based on ASOs (Fire et al. 1998). While ASOs are not compatible with pooled screening, nevertheless they have become an indispensable tool for validating screen results. ASOs are short single-stranded oligonucleotides (13–25nt) that are chemically modified to achieve stability and potency (Dias and Stein 2002). ASOs hybridise by sequence complementarity to cellular RNAs and activate degradation by the enzyme RNase H (Crooke 2017) (Fig. 1c). ASOs display low off-target effects and are appropriate for use in humans for therapeutic applications (Crooke et al. 2021). Further advantages are their ability to be delivered into cells without the need of a delivery vehicle (“free uptake”), and particularly important for lncRNAs, they appear to degrade nascent RNAs in the process of transcription, thus accessing nuclear target populations (Pallarès-Albanell et al. 2019). However, due to the difficulty in designing effective on-target ASOs (typically around 40% are effective), the lower uptake efficiency when compared with vehicle mediated delivery methods (Stein et al. 2010; Hs et al. 2012) and the cost of their chemical synthesis, so far just one ASO screen for growth-modulating lncRNAs has been reported to date (Ramilowski et al. 2020).

#### Perturbation approaches: CRISPR

The advent of CRISPR genome editing has profoundly impacted the field of lncRNA functional genomics. For the first time, researchers have an effective tool that can be adapted to a variety of perturbations (repression, silencing, activation), can be targeted to the gene locus or the RNA product, displays reduced off-target effects, and most importantly, can be conveniently scaled to high throughputs (Sanson et al. 2018; Doench et al. 2016; Zhu et al. 2016; Diao et al. 2017; Gasperini et al. 2017).

CRISPR comprises an RNA:protein complex. The single guide RNA (sgRNA) consists of a 20 nt variable RNA sequence or “spacer”, which recognises by homology a specific genomic site followed by a protospacer adjacent motif (PAM) (Jinek et al. 2012; Mali et al. 2013; Cong et al. 2013; Sander and Joung 2014). The spacer is fused to an invariant structured “scaffold” that is recognised by the Cas9 protein. Researchers may target this complex to desired regions by simply identifying a PAM in that region, and designing the spacer sequence to recognise the adjacent 20mer. In turn, the Cas9 protein “cargo” may be engineered to perform various tasks at its destination, from DNA endonucleolytic cleavage in its wild-type form, to catalytically dead mutants (dCas9) fused to a growing array of effector domains (Fig. 1b) (Qi et al. 2013; Gilbert et al. 2013; Dominguez et al. 2016). Fusions carrying transcriptional inhibitor or activator domains form the basis for CRISPR inhibition (CRISPRi) and CRISPR activation (CRISPRa), respectively (Gilbert et al. 2013). This programmability enables CRISPR to be rapidly deployed for a wide range of desired perturbations (Doench et al. 2016). Because sgRNAs can be delivered by lentiviral vectors (Kosicki et al. 2018), CRISPR perturbations enable almost unlimited scalability in pooled screening format. Together these features make CRISPR a versatile and useful tool for discovering functional lncRNAs.

Nonetheless, CRISPR does present a number of hurdles that must be overcome. First of all, Cas9 is a bacterial protein and thus is highly immunogenic (Charlesworth et al. 2019). Induced Cas9 systems can help to mitigate this harmful effect in cells. WTCas9 nuclease activity results in double strand breaks (DSBs) that can cause genome rearrangements and cell death (Chapman et al. 2012; Kosicki et al. 2018; Leibowitz et al. 2021). The latter effect is stronger in cells expressing P53 (Bowden et al. 2020). The outcome is that sgRNAs may lead to non-specific apoptosis caused by the technique itself and not by the effect of the CRISPR modification. This must be addressed in screens by the careful design of phenotypically neutral controls: sgRNAs targeting intergenic regions give a better indication of background including DSB toxicity, rather than (often used) non-targeting controls, such as scrambled sequences, which will not cause DSBs and can lead to false positive hits (Aguirre et al. 2016; Haapaniemi et al. 2018). A second issue is off-targeting: while far lower than for shRNA (Smith et al. 2008), many sgRNAs do recognise non-targeted sites at non-zero frequency, resulting in off-target effects (Zhang et al. 2015). These effects can be largely avoided by careful sgRNA design using strict off-target filtering (Shalem et al. 2015). Higher concentrations of Cas9/sgRNA can lead to increased off-targets rates (Wu et al. 2014), therefore it is necessary to control for these concentration when performing in vivo experiments.

The first means of perturbing lncRNAs by CRISPR harnesses the ability of wild-type Cas9 to generate DSBs. This approach requires an understanding of the cellular processes that repair the resulting DSBs. The most prevalent pathway is non-homologous end joining (NHEJ), which is a non-templated method that repairs breaks but often introduces untemplated insertions and deletions (indels) at the repair site (Ceccaldi et al. 2016). These properties proved highly useful for knocking out PCGs, since sgRNAs targeted to open reading frames (ORFs) generate frameshift mutations that scramble the encoded peptide sequence (Shalem et al. 2015). Because, by definition, lncRNAs contain no encoded peptide, it is uncertain whether small indels are sufficient to impact lncRNA activity. Therefore, loss of function by CRISPR calls for more elaborate strategies. The most frequent approach is “CRISPR deletion” (CRISPR-del), where two wild-type CRISPR-Cas9 complexes are recruited to sites flanking a targeted genomic region (Aparicio-Prat et al. 2015). Simultaneous NHEJ gives rise to genomic deletion. Efficiency tends to lie in the range 40–60% of alleles (Gasperini et al. 2017; Aparicio-Prat et al. 2015; Kraft et al. 2015; Ran et al. 2013; Canver et al. 2014; Vidigal and Ventura 2015; Antoniani et al. 2018; Pulido-Quetglas et al. 2017), although often much less, and these rates broadly decline with the size of the deleted region(Canver et al. 2014).

CRISPR-del may be employed for lncRNA loss of function in several ways. The first and most obvious is by deletion of the entire gene body (Durruthy-Durruthy et al. 2015). However, this strategy entails several drawbacks. LncRNA genes can span several hundred kilobases. Such deletions tend to have low efficiency (Canver et al. 2014), and may well remove other overlapping functional elements, including PCGs and enhancers and thereby lead to false positive phenotypes. Removal of lncRNA TSS via targeted deletion of ~ 0.5 to 5 kb is a more practical alternative, by reducing the length of the deletion to a few hundreds to thousands of bases, increasing efficiency and uniformity, and decreasing the chance of deleting unrelated elements (Zhu et al. 2016; Pulido-Quetglas et al. 2017; Lavalou et al. 2019). Even effective deletions may not result in hoped for loss of gene expression: compensatory promoter activation has been reported in some cases (Lavalou et al. 2019). Given the deletion size mentioned, the TSS deletion strategy requires accuracy of lncRNA annotations at the 5′ end with a resolution of ~ 1 kb.

Other flavours of CRISPR can perturb lncRNA expression without permanently mutating DNA. By engineering appropriate fusion proteins with catalytically dead Cas9 (dCas9), one may achieve gene activation (CRISPRa) or inhibition (CRISPRi) (Liu et al. 2017; Horlbeck et al. 2016). Importantly, both these technologies require recruitment to a rather small window of ~ 200 bp with respect to the TSS, making them highly sensitive to accurate TSS annotation (Sanson et al. 2018). Resulting chromatin reorganisation of both methods can have and indirect effect on neighbouring genes. CRISPRa mechanism is capable of open the chromatin and allows transcription machinery to access genes located nearby the targeted region increasing their expression. Similarly, indirect reduction in gene expression can be observed when targeting genes with CRISPRi (Horlbeck et al. 2016; Groner et al. 2010). Researchers should therefore validate the results obtained by these methods analysing any unintended changes in expression of nearby genes. These approaches avoid issues of DSB toxicity, while having the additional benefit of being compatible with a variety of inducible systems, affording the researcher temporal control over gene perturbations (Sun et al. 2019).

#### Pooled screening

Genetic screens are a powerful method to test the effect of gene perturbations in a high-throughput way (Sanson et al. 2018; Doench et al. 2016; Zhu et al. 2016; Liu et al. 2017). Screens in cultured cells can be performed in two formats: arrayed and pooled. Arrayed screens apply a single perturbation to multiple cells in one well. They require robotics equipment, due to the large number of wells involved, and they require synthesis of many individual perturbation reagents (siRNAs or sgRNAs) (Lord et al. 2008; Whitehurst et al. 2007). Screen results are read out from each individual well, and as a result are relatively unconstrained in terms of the phenotypic features that can be measured, extending to microscopy and image analysis (Stojic et al. 2020).

Pooled screens, in contrast, involve introducing a mixed pool of perturbation constructs into a single cell population (Fig. 1d). Libraries are synthesised as a mixture using increasingly inexpensive oligonucleotide “megasynthesis” (Doench 2017), and delivered with genomically integrating lentiviruses (Sanson et al. 2018). Viruses are usually applied at low titres (multiplicity of infection, MOI, ~ 0.3), so that every cell in the population carries one perturbation. A selection is applied in order to isolate two or more cell populations with different phenotypes. Genomically integrated perturbation sequences, usually sgRNAs, are then used as barcodes to determine the differences in library composition between cell populations of different phenotypes, and hence infer functional lncRNAs contributing to said phenotypes (Zhu et al. 2019; Boettcher et al. 2019). This highlights the key constraint of pooled screening: phenotypic readouts are restricted to those which can be sorted in some way (Sanson et al. 2018; Shalem et al. 2015). These include cell fitness/proliferation, fluorescence, survival in response to insult, or migration(Sanson et al. 2018; Zhu et al. 2016; Shalem et al. 2015; Liu et al. 2018), but rules out imaging-based readouts.

Even with the requirements of pooled screens—i.e. phenotypic selection, next generation sequencing, and deconvolution of the data to determine perturbation abundances—the benefits these screens provide compared to arrayed screens are significant. In pooled screens, libraries can be created, delivered to cells and analysed as a single sample, considerably reducing the cost and hands-on time. This also avoids the capital investment and training required for robotics necessary for arrayed screening. The fact that only a single sample has to be analysed, helps to reduce batch effects and increases the statistical power of the analysis, since all perturbations, tests and controls, are treated with the same exact conditions. These advantages have led to growing adoption of pooled CRISPR screens.

**Fig. 2:**
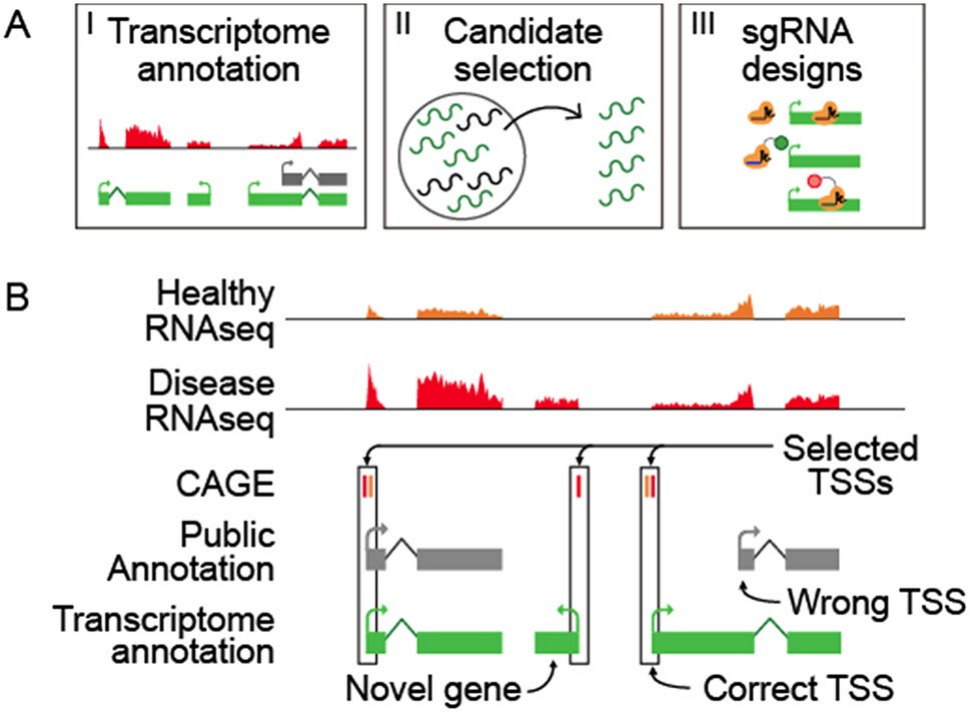
Accurate annotations for CRISPR screens. **a** The principal steps in custom pooled screening library design. **b** Refining the annotation of lncRNA transcription start sites (TSS) for library design

A key requirement of pooled screens is the screening library. Libraries targeting all or subsets of PCGs are rapidly growing in quality, and are available from multiple suppliers (Doench et al. 2016). In contrast, few such resources are presently available for lncRNAs, due to a number of factors. Firstly, the number and quality of lncRNA annotations increases so rapidly that libraries rapidly become obsolete. Secondly, lncRNAs have highly cell-type-specific expression profiles, meaning that available libraries designed for a given purpose, may not cover a useful proportion of targets in a different biological assay or cellular background. These factors mean that researchers are likely to have to design custom lncRNA screening libraries for the immediate future. Provide a uniform guideline is the purpose of the present Review. The process of designing a screening library can be broken into three principle steps: gene annotation, candidate selection, and sgRNA design (Fig. 2a). These steps are explained in more detail in the following sections.

## Accurate transcript annotations

Gene perturbation, particularly by CRISPR, depends critically on recruiting Cas9 to a narrowly defined window around the TSS (Sanson et al. 2018). Consequently, accurate maps of gene and transcript structures are essential for functional screening (Sanson et al. 2018; Bergadà-Pijuan et al. 2020). These maps are referred to as annotations, and specify the exact location of gene’s constituent transcripts, introns and exons (Harrow et al. 2006). Most importantly in the present context, annotations record the expected location of TSSs, being simply the start position of the first exon for the transcript(s) comprising a gene (Fig. 2b).

Despite their importance, lncRNA annotations remain an imperfect reflection of the underlying biological reality, and are best regarded as work in progress, provided by several different sources and created with different approaches (Uszczynska-Ratajczak et al. 2018). GENCODE, for example, provides lncRNA annotations for ENSEMBL and is a mixture of manual and high-quality experimental annotations, which ensures a good quality but relatively small size and incomplete coverage for many cell types (Uszczynska-Ratajczak et al. 2018; Derrien et al. 2012; Lagarde et al. 2017). This and the other principle manually curated resource, RefSeq, have formed the basis for several shRNA and CRISPR screen designs for human and mouse genomes (Lin et al. 2014; Beermann et al. 2018; Zhu et al. 2016). A more complete and detailed lists of resources for lncRNA annotations can be found in two reviews: Uszczynska-Ratajczak et al. (Uszczynska-Ratajczak et al. 2018) and Richard et al. (Charles Richard and Eichhorn 2018).

An important drawback of the above public annotations, is that they are not *comprehensive*—they omit many genuine lncRNAs (Uszczynska-Ratajczak et al. 2018). This may occur due to the distinct annotation protocols and criteria employed. However, another important cause is the fact that annotations are based on published transcriptomic resources, or from focussed studies in a small number of cell types (Lagarde et al. 2017). Thus, the lncRNAs they contain are biassed towards those expressed in widely studied cell lines and organs. This will impact researchers who wish to perform a screen in any cell model that is not well represented in the above datasets.

Two solutions are available to the researcher to address this lack of annotation comprehensiveness in their model of interest. The first is to merge several public annotations into a single, larger and more comprehensive one. Several software packages are available for this (Trapnell et al. 2010; Pertea et al. 2015). A second, more time-consuming but more effective approach, is to create a custom annotation through transcriptome assembly (Grabherr et al. 2011; Kovaka et al. 2019; Hölzer and Marz 2019). By using RNA-sequencing data from the cell model of interest, a new “assembly” of transcriptome annotation can be built bioinformatically (Liu et al. 2020). The advantage here is that the assembly reflects the transcriptome in the cells where the screen is to be performed. Thus, it might contain many novel and cell specifically expressed lncRNAs that are missing in public annotations (Roberts et al. 2011). Novel assemblies are usually further merged with public assemblies for extra confidence (Joaquina Delás et al. 2017; Liu et al. 2017). Transcriptome assemblies are algorithmically predicted from short RNA-sequencing fragments and therefore they might not be 100% accurate. In future, this gap can be mitigated by using long read RNAseq data, which captures the full sequence of lncRNA transcripts and the assembly step will not be necessary (Lagarde et al. 2017).

A second key feature of annotations is their *completeness*—or whether they accurately record the location of the TSS (Uszczynska-Ratajczak et al. 2018). LncRNAs can present multiple TSSs and correct identification is crucial (Mattioli et al. 2019; Kindgren et al. 2018). As mentioned before, CRISPR perturbations depend on recruitment to a small window around the TSS, meaning that even minor inaccuracies in TSS annotation may result in false negative results. Unfortunately, lncRNA annotations are poor at correctly recording TSS locations, as defined by gold-standard evidence from Cap Analysis of Gene Expression (CAGE), a sensitive method to map 5′ ends of transcripts (Uszczynska-Ratajczak et al. 2018; Hon et al. 2017). Although transcriptome assemblies have particularly poor performance at identifying TSS (Lagarde et al. 2017) the FANTOM group has accurately re-annotated lncRNA TSSs from multiple transcripts collections by including CAGE analysis into the analysis (Hon et al. 2017).

Both of these issues with lncRNA annotations, missing genes or incompleteness at 5′ end, will ultimately result in false negatives. This was demonstrated recently by reanalysis of published CRISPRi screens, where it was found that lncRNAs are significantly less likely to be hits when their TSS is inaccurately annotated (as judged by CAGE data) (Bergadà-Pijuan et al. 2020).

Another key variable for researchers is the species under study. Despite their drawbacks, lncRNA annotations in human and mouse are far more advanced than other model organisms (Sundaram et al. 2017). Less is known about lncRNA populations in non-model species, although we have no reason to believe they are any less important or numerous. Researchers working on non-model species will, given their lack of lncRNA annotations, have to rely even more on transcriptome assemblies for library design.

## Narrowing down the best candidates

The number of genes that can be included in a screen is limited by cost and other practical parameters. A typical CRISPR screen requires multiple sgRNAs per target gene (usually ~ 10), a coverage of 100–1000 individual cells per sgRNA sequence, and 100 s of NGS reads per sgRNA (Sanson et al. 2018; Doench 2017). Therefore, materials cost increases with the number of candidates tested. Fortunately, it is not necessary to screen the entire population of 100,000 + annotated lncRNAs (Fang et al. 2018), because only a small subset are present in a given cell model. Thus, the second step of library design involves filtering to focus on a reduced set of candidate lncRNAs that are most likely to contain screen hits. More so than the other two steps, this one is most specific to the particular biological system under study and requires the greatest amount of user discretion.

**Fig. 3:**
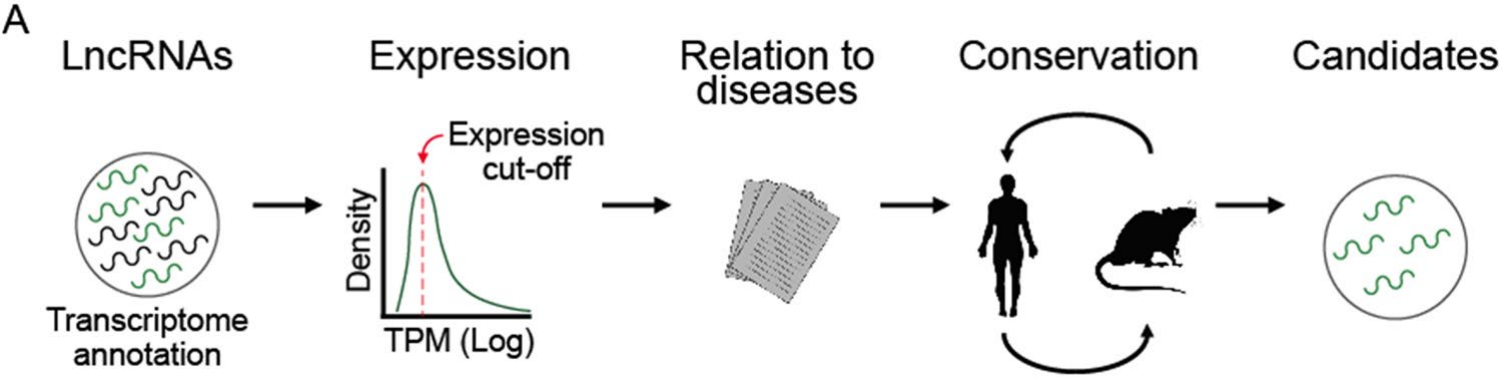
Selection of screen candidates. **a** Schematic representation of possible filters to apply for candidate selection for screens

Several filtering methods can be applied in order to enrich the final list of candidates for likely hits (Fig. 3a). The primary and most obvious filter is expression in the cells of interest. In principle, only expressed transcripts should be biologically active, and the majority of silent lncRNA genes can be omitted. Thus, it will be necessary to quantify specific RNAseq data from the screen model to select those lncRNAs expressed. For example, in Liu et al. only ENSEMBL lncRNAs expressed in the cell lines used in the study were included in the screen (Liu et al. 2018). Due to the low expression levels of lncRNAs, thresholds as low as 0.1 transcripts per million (TPM) can be required to not miss any relevant lncRNAs, especially given the exceedingly low expression observed for some functional lncRNAs (Seiler et al. 2017). This step alone will substantially narrow the candidate set, and indeed may alone be sufficient to reach the desired library size.

Another important consideration for candidate selection is gene copy number. When the goal is a complete knockout, it will be more challenging to achieve for genes present at > 2 copies per cell. Furthermore, targeting these genes with CRISPR will generate multiple DSBs, increasing the likelihood of non-specific toxicity to the cell (Aguirre et al. 2016). Information for the gene copy number in multiple cell lines can be obtained from the Cancer Cell Line Encyclopedia (CCLE; https://sites.broadinstitute.org/ccle/). These considerations are further complicated by the fact that oncogenes are frequently amplified in tumours, meaning that phenotypic effects of targeting oncogenic lncRNAs may be a mixture of both specific and non-specific effects. To our knowledge, this issue remains to be satisfactorily resolved, apart from careful validation by ASOs or other DSB-independent perturbations. Differential expression can also be used as a method for selection. For example, when screening for lncRNAs involved in cancer development, tumor samples can be compared against its healthy counterpart to find tumour-upregulated lncRNAs (Zhu et al. 2016).

Some of the filters will be already imposed by the screen method itself. For example, if we use a CRISPR deletion approach, only intergenic (non-PCG overlapping) lncRNAs might be targeted, so as to avoid perturbation of a nearby PCG. In this case a minimum distance from the TSS of lncRNAs to the nearest PCG can be applied as a filter.

Many lncRNAs have been associated with diseases. Several online databases have compiled such lncRNAs, and may be used as a valuable filter for candidate selection (Vancura et al. 2021; Bao et al. 2019; Wang et al. 2019; Zhao et al. 2020). The drawback of this approach is that it will omit novel lncRNAs from transcriptome assemblies. Other useful evidence for lncRNA function in disease may come from germline variants lying nearby (Giral et al. 2018; Aznaourova et al. 2020) (or better, that are also quantitative expression trait loci or eQTLs) (Goede et al. 2021). Similarly, somatic single nucleotide variants or copy number variants are also important evidence for prioritising cancer lncRNAs (Lanzós et al. 2017; Minotti et al. 2018; Gao et al. 2019). The latter datasets (with the exception of eQTLs) have the added benefit of being compatible with novel transcriptome assemblies.

LncRNAs are evolutionarily less conserved than PCGs (Uszczynska-Ratajczak et al. 2018). However, the conservation of their exon structure and expression pattern in different developmental stages across related species is important evidence for functionality (Chodroff et al. 2010; Hezroni et al. 2015; Sarropoulos et al. 2019; Carlevaro-Fita et al. 2020). Although conservation is not a limitation (Ruan et al. 2020), the presence of orthologues in other species can be used as a filter for screen candidates.

After filtering and selecting the optimal lncRNA candidates only one step remains: the design of an optimised library of perturbation constructs.

## Designing sgRNA libraries

CRISPR perturbation efficiency is directly linked to sgRNA design. Improved designs will increase the performance of the screen and avoid false negatives. The 20 nt sgRNA spacer sequence will dictate the on-target activity and the number of possible off-target regions (Doench et al. 2016; Abadi et al. 2017; Liu et al. 2020). Although sgRNA sequence itself and folding stability are key to increase the efficiency for the on-target region, orientation of the guide in relation to the target gene has also been reported to impact sgRNA efficiency (Wang et al. 2014). Various algorithms and tools are available to design and calculate on-target efficiency of sgRNAs from query sequences or gene IDs (Doench et al. 2016; Horlbeck et al. 2016; Xu et al. 2015; Wong et al. 2015; Concordet and Haeussler 2018). One of these algorithms also account for CRISPRi/a designs (Sanson et al. 2018; Doench et al. 2016). Of relevance for lncRNAs screens, tools for paired sgRNA designs are also available (Pulido-Quetglas et al. 2017; Perez et al. 2017). An important drawback of tools accepting only gene IDs, is that sgRNAs can only be designed for known genes, leaving novel genes and non-genic regions untargetable. Some tools only provide sgRNA designs for one or a limited number of targets at a time, which makes them unsuitable for scaling up to high throughputs, while others can provide designs for an unlimited number of target regions (Pulido-Quetglas et al. 2017) (https://portals.broadinstitute.org/gppx/crispick/public). A summary of these tools can be found in Table 1.

**Table 1:** Summary of sgRNA design tools referenced in this review

Tool name	Design type	Limitation	Link	References
CRISPick	ko/i/a	500 gene IDs	https://portals.broadinstitute.org/gppx/crispick/public	Doench et al. (2016)
CRISPETa	ko	–	http://crispeta.crg.eu/	Pulido-Quetglas et al. (2017)
SCC	ko/i/a	Sequence length < 10,000 bp	http://crispr.dfci.harvard.edu/SSC	Xu et al. (2015)
WU-CRISPR	ko	1 sequence; 26–30 k bp	http://crisprdb.org/wu-crispr/	Wong et al. (2015)
CRISPOR	ko	1 sequence: < 2300 bp	http://crispor.tefor.net/	Concordet and Haeussler (2018)
GuideScan	ko	–	http://www.guidescan.com/	Perez et al. (2017)

**Fig. 4:**
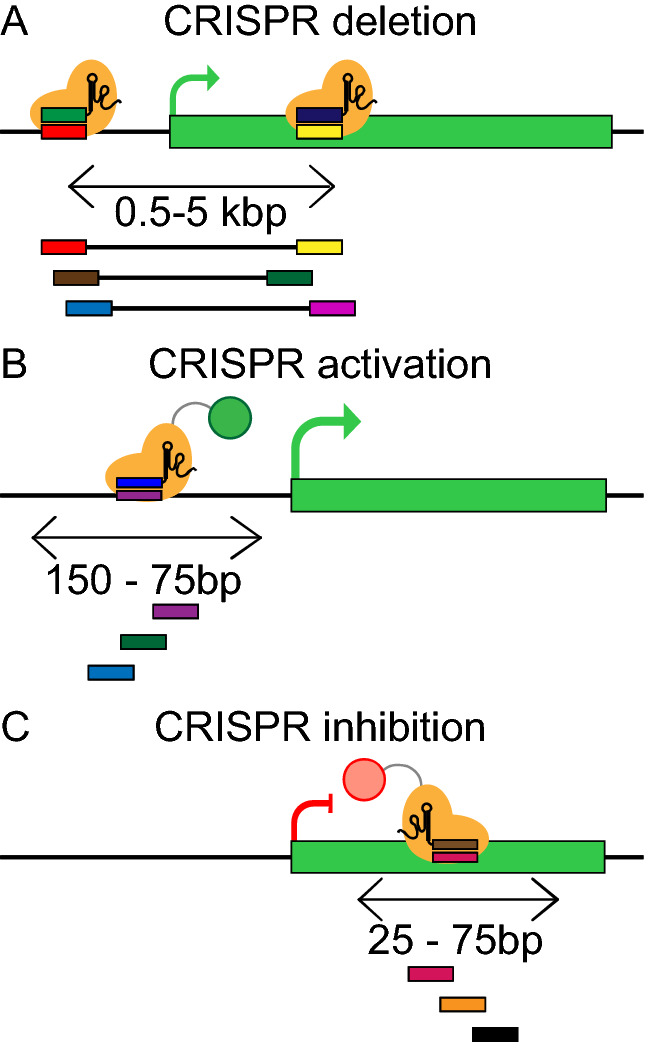
Optimal sgRNA design for diverse CRISPR perturbations. Optimal locations of paired sgRNAs for **a** TSS deletion, **b** CRISPR activation, and **c** CRISPR inhibition. It is recommended to design at least three sgRNAs per target site

Genomic regions with complementarity to the sgRNAs can cause undesired off-target effects. Off-target regions can tolerate mismatches, particularly when they fall more distal to the PAM end of the sgRNA-DNA hybrid (Hsu et al. 2016). Removing sgRNAs with potential off-target matches is routinely performed in library designs (Doench et al. 2016). Several online tools are also available to find off-target regions and calculate their scores (Doench et al. 2016; Bae et al. 2014; Stemmer et al. 2015). Different scoring algorithms will rank sgRNAs differently, thus, concordance between predicted and measured activity of the guide can vary (Labuhn et al. 2018). To mitigate the fluctuation of efficiency and to increase statistical power, it is common practice to design several sgRNA (4–10) per target region (Bodapati et al. 2020) (Fig. 4). This number can be reduced to two with optimal sgRNAs targeting known essential control genes (Wong et al. 2015). While broadly used genome-wide Cas9 libraries targeting PCGs have on average 10^5^ sgRNAs, this number is halved for lncRNA targeting CRISPR screens.

Not only the characteristics of the sgRNA are important but also the location of the on-target regions. For example, in CRISPR deletion screens, the distance between the two sgRNAs will have an impact on the efficiency. Although large deletions have been achieved (Mizuno-Iijima et al. 2020) efficiency decreases for deletions larger than 0.5—5 k bps (Fig. 4a) (Zhu et al. 2016; Canver et al. 2014; Han et al. 2014; Zheng et al. 2014). CRISPRi and CRISPRa efficiency also depends on the distance of the on-target region to the targeted TSS. Optimal sgRNA design ranges for this approaches are extremely narrow, lying between + 25 to + 75nts downstream of the TSS for CRISPRi and − 150 to − 75 nts upstream of the TSS for CRISPRa (Sanson et al. 2018; Bergadà-Pijuan et al. 2020; Radzisheuskaya et al. 2016) (Fig. 4b and c). CAGE data can be used to select optimal transcript TSSs to optimise CRISPRi/a designs (Sanson et al. 2018).

Positive and negative controls are crucial to properly analyse the performance of the library and to measure the CRISPR perturbation effect. A total of at least 300 sgRNAs targeting positive control genes are needed to effectively control the false discovery rate (Bodapati et al. 2020). Genes known to influence the screening phenotype are typically used as positive controls (Aguirre et al. 2016; Haapaniemi et al. 2018). Essential genes, such as those encoding ribosomal proteins, or growth-promoting genes, are frequently employed as positive controls in CRISPR screens based on cell fitness/proliferation (Zhu et al. 2016; Liu et al. 2018), as their sgRNAs should disappear or “drop out” in the final population of cells. A minimum of three sgRNAs should be used to target these controls. Negative controls (sometimes referred as neutral controls) are not expected to influence phenotype and are used as a reference with which to identify screen hits. Intergenic regions (Zhu et al. 2016) or the Adeno-Associated Virus Integration Site 1 (AAVS1) where deletions have been proved non-deleterious (Smith et al. 2008; Chu et al. 2015; Hayashi et al. 2020) are good choices for design of negative control sgRNAs (Zhu et al. 2016; Liu et al. 2018). In experiments with wild-type Cas9, we recommend the use of targeting negative controls (i.e. that target a non-functional genomic region) rather than non-targeting controls (i.e. containing a spacer with no genomic match), since the former more accurately model the non-specific toxicity arising from DSBs.

## Outlook

The discovery of functional lncRNAs has been revolutionised by pooled screening technology, particularly that implemented with CRISPR and its variants. CRISPR screening is capable of functionally interrogating thousands of lncRNAs in a single experiment, without the overheads associated with arrayed screening. Its favourable performance across multiple features (reduced off-targets, high on-target efficiency, flexible delivery method and high programmability) has led to its rapid adoption over RNAi-based approaches. As the volume of CRISPR data increases, further improvements are likely in aspects such as sgRNA on-target and off-target activity.

Space constraints meant that we could not discuss an upcoming variation of CRISPR screening based on direct RNA perturbation with Cas13 and other enzymes (Anton et al. 2018; Cox et al. 2017; Abudayyeh et al. 2017; Xu et al. 2020). Instead of targeting the gene, RNA-targeted CRISPR directly destabilises or otherwise perturbs the RNA transcript itself. Although it is still in development, several publications have already demonstrated its efficiency in different organisms (Yang et al. 2019; Kushawah et al. 2020; Huynh et al. 2020). Similar to CRISPRi/a, Cas13 can be converted into a programmable RNA binding platform by mutating its catalytic site (dCas13) and fusing it to a catalytic enzyme with desired activities. In this way, dCas13 could be use, for example, as a tool for live cell RNA imaging (Palaz et al. 2021). This may be a promising option for gene therapy applications, where DNA mutation is undesirable (Anton et al. 2020).

The practicality and versatility of CRISPR screening makes it capable of identifying lncRNAs mediating a wide variety of cellular processes in healthy and diseased biological contexts. We expect that as annotations improve and screen components become standardised, this approach will become increasingly widely used to identify molecular components and therapeutic targets among the tens of thousands of uncharacterised lncRNA genes.
